# Is mandibular posterior dento-alveolar intrusion essential in treatment of skeletal open bite in adult patients? A single center randomized clinical trial

**DOI:** 10.1186/s12903-025-05778-w

**Published:** 2025-04-07

**Authors:** Heba E. Akl, Amr M. Abouelezz, Fouad A. El Sharaby, Mohamed Abd-El-Ghafour, Amr R. El-Beialy

**Affiliations:** 1 Department of Orthodontics, Faculty of Dentistry, Cairo University, Cairo, Egypt; 2 Cleft-Craniofacial Orthodontist, Member, American Cleft Palate-Craniofacial Association (ACPA), Royal College of Surgeons of Edinburgh, Member, UK

**Keywords:** Open bite, Molar intrusion, Posterior segment intrusion, Miniscrews, Skeletal anchorage

## Abstract

**Background:**

Anterior open bite (AOB) malocclusion usually represents a complicated and advanced orthodontic problem. The skeletal variant of AOB used to be treated with a combined orthodontic and surgical approach, until the posterior segments’ intrusion has been validated as an alternative, effective and conservative treatment modality for such cases with comparable outcomes to the surgical approach. The objective of this two-arm parallel randomized clinical trial was to compare the effects of mini-screw supported maxillary versus bi-maxillary buccal segments’ intrusion on the amount of anterior open bite closure.

**Methods:**

Twenty-two adult patients aged 17–25 years, with skeletal open bite and anterior dental separation of 3–8 mm were randomized to either the comparator (Maxillary Intrusion with Consolidation of mandibular buccal segments-MIC) or intervention (Bimaxillary buccal segments’ intrusion-BMI) groups. Miniscrew-assisted buccal segments’ intrusion was instituted using fixed appliances on rigid stainless steel archwires (19 × 25 stainless steel) via nickel-titanium coil springs in the maxilla and memory chains in the mandible. The intrusion force was 200 g per maxillary buccal segment in both groups, and it was 150 g for each mandibular posterior segment in the BMI group. Duration of intrusion was 6 months.

**Results:**

Anterior open bite was significantly closed in both groups with means of 3.8 ± 0.84 (95% confidence interval [CI] 3.2–4.4) and 3.84 ± 1.47 mm (CI;2.8–4.9) for the MIC and BMI groups, respectively with no significant difference between them (p-value < 0.05). Maxillary posterior teeth experienced significant intrusion in both groups, with a mean of 2.89 ± 1.13 mm (CI;2.63–3.14) in the MIC group and 2.26 ± 1.62 mm (CI;1.89–2.62) in the BMI group. Statistically significant mandibular posterior teeth intrusion occurred in both groups with means of 0.86 ± 0.91 (CI;0.65–1.06) and 0.33 ± 0.84 mm (CI;0.14–0.52) in the BMI and MIC groups, respectively, with a statistically significant difference of 0.53 ± 0.14 (CI;0.25–0.8) mm. However, such difference was considered clinically insignificant.

**Conclusions:**

Anterior open bite closure could be successfully achieved with maxillary buccal segments intrusion without the need for active intrusion of the mandibular posterior segments, as long as the latter are efficiently consolidated.

**Trial registration:**

The trial was prospectively registered at clinicaltrials.gov with an identifier number of NCT04713280.

## Background

One of the most challenging problems encountered in the orthodontic practice is the skeletal open bite (SOB) malocclusion [1, 2]. The co-occurrence of multiple etiologies and involvement of skeletal, dento-alveolar and soft tissue components may underly the complexity of those cases [2, 3]. 

Over-eruption of maxillary and/or mandibular posterior segments has been accused of contributing to the features of SOB [4, 5], resulting in clockwise mandibular rotation, increased lower anterior facial height, profile convexity and lip incompetence [6, 7]. Several decades ago, the gold standard treatment for SOB in adult patients was the combined surgical and orthodontic approach [8, 9]. However, the increased use of skeletal anchorage has allowed conservative and effective anterior open bite (AOB) closure via posterior teeth intrusion [10, 11], with outcomes that are comparable to those of the surgical intervention [9, 12]. Despite such success, the available evidence from systematic reviews reports lack of high-quality, methodologically sound trials investigating the buccal segments’ intrusion for AOB closure, in terms of indications, limitations, outcomes and stability [8, 12–14]. 

A frequently reported finding during maxillary buccal segments’ intrusion was the over-eruption of the mandibular buccal segments [10, 15] which resulted in an unsatisfactory amount of AOB closure [10]. ***Xun et al.*** [16] achieved 4.2 mm of AOB closure by intruding both maxillary and mandibular buccal segments, while ***Akl et al.*** [10] achieved 2.2 mm only for the same outcome utilizing maxillary buccal segments’ intrusion alone. Therefore, it was a plausible assumption that active intrusion of the mandibular buccal segments in addition to the maxillary buccal segments would yield greater amounts of AOB closure.

Hence, the aim of this study was to investigate the effect of maxillary buccal segments’ intrusion with consolidation versus active intrusion of the mandibular posterior segments on the amount of AOB closure in a randomized clinical trial methodology.

## Methods

### Trial design

The study was a two-arm parallel randomized clinical trial with an allocation ratio of 1:1. No changes were done after commencement of the study. Prospective trial registration at clinical trials.gov was done on 19/01/2021 with an identifier number NCT04713280. The updated CONSORT guidelines [17] for reporting parallel group randomized trials were followed.

### Participants, eligibility criteria, and settings

The study was carried out in the clinic of the Orthodontic Department-Faculty of Dentistry Cairo University, after approval of the Centre of Evidence Based Dentistry and Research Ethics Committee. All subjects were informed about the study purpose and procedures and signed informed consents. Patients’ eligibility criteria are demonstrated in Table 1.

**Table 1:** Patients’ eligibility criteria

Inclusion criteria	Exclusion criteria
Adult patients aged 17 to 25 years.	Open bite due to under-eruption of anterior teeth, without skeletal components.
Skeletal open bite as measured on lateral cephalograms (MMA>32°, SNMP> 37°).	Decreased incisal show or reversed smile arc, indicating the need for incisors’ extrusion.
Skeletal class 1 or mild to moderate skeletal class 2, only mild skeletal class 3.	Severe skeletal class 2 and moderate to severe class 3 that are indicated for extractions and/or orthognathic surgery.
Dental open bite ranging between 3–8 mm.	Moderate to severe crowding that would necessitate teeth extraction
Normal incisal show at rest and on smiling.	Medically compromised patients, or those using chronic medications that might affect the extent or rate of tooth movement.
Full set of maxillary and mandibular posterior teeth.	

### Interventions

#### Preparatory phase

Patients were randomly assigned to either the comparator group, who received maxillary posterior segments’ intrusion with consolidation of the mandibular buccal segments (MIC), or the intervention group, who had bimaxillary intrusion (BMI).

A Segmented fixed appliance (*American Orthodontics*,* Washington*,* USA-Mini Master Roth 022 Orthodontic brackets*) was installed on both maxillary and mandibular posterior segments from first premolar to second molar in all patients. A lingual arch was placed on mandibular first molars, and was relieved by 3 mm from the lingual surfaces of lower incisors [16]. For the intervention group, buccal root torque was added in the lingual arch before cementation. Levelling and alignment of the posterior segments was done in the four quadrants till a rigid stainless steel archwire of gauge 0.019 × 0.025” was reached.

**Fig. 1:**
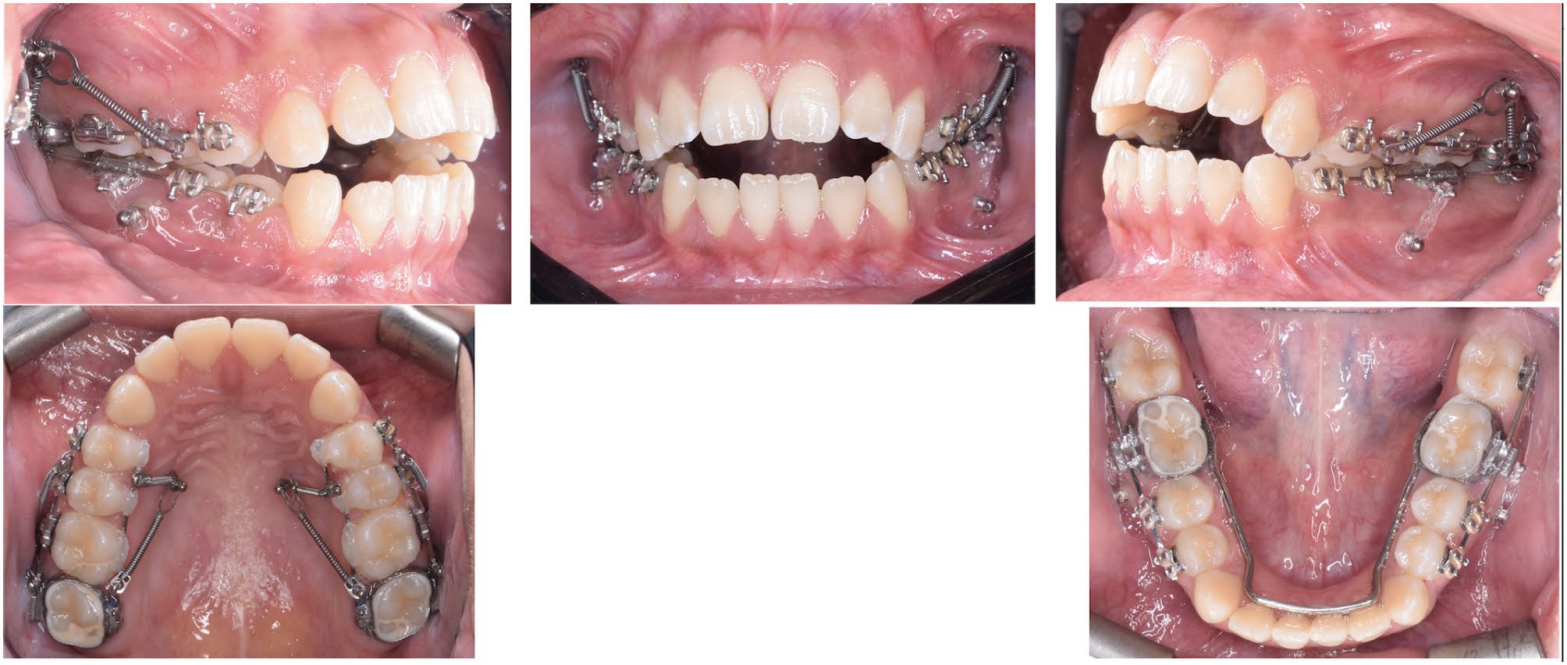
The appliance assembly in a patient of the BMI group, showing 6 miniscrews, closed NiTi coil springs in the upper arch and memory chains in the lower arch

Patients of both groups received infra-zygomatic (*3 M Unitek TAD*, 1.8*10 mm) and palatal (*3 M Unitek TAD*, 1.8*8 mm) miniscrews. The palatal screws were placed between the first and second premolars 8–10 mm from the gingival margin. Hence, each patient received 4 miniscrews for maxillary posterior dento-alveolar intrusion. Patients in BMI group received 2 additional miniscrews in the mandibular buccal area between second premolars and first molars (*3 M Unitek TAD*, 1.8*8 mm) for active intrusion of the mandibular posterior segments (Fig. 1). Patients were then sent to take the pre-intrusion CBCT (Planmeca ProMax 3D Mid with large field of view 20 cm * 17 cm,* 90 Kvp and 8 mA).*

#### Intrusion phase

Bonding of upper palatal intrusion wires was done using composite restorative material (*3 M ESPE -St.Paul*,* MN*,* USA*). The pre-intrusion CBCT of each patient was used to calculate the force to be applied by the long coil springs (anterior buccal and posterior palatal) as described in a previous study in order to deliver the required amount of intrusive force (50 g), aiming at bodily intrusion of the segments [10]. Closed Nickel titanium (Ni-Ti) coil springs were used to deliver a 200 g of net intrusive force for each maxillary posterior segment in both groups (50 g by each coil spring) [10]. Memory chains were used to apply a 150 g of intrusive force for each mandibular posterior segment in the BMI group [16]. Patients were followed up every 2 weeks to measure the anterior open bite clinically, check the appliance integrity, re-calibrate the Ni-Ti coil springs (every 4 weeks using *CORREX force gauge 50–500 g*,* Dentaurum*,* Germany)* and place new memory chains in the BMI group patients. The duration of intrusion was 6 months, after which patients took the post-intrusion CBCT.

#### Miniscrew failure and potential harms

The stability of miniscrews was checked in each follow-up visit (every 2 weeks). The mini-screw was considered failed when it was mobile that it could not withstand the applied orthodontic force through the respective Ni-Ti coil spring or memory chain [18]. Any inflammation or soft tissue hyperplasia was reported and the patient was instructed to do strict oral hygiene measures to avoid mini-screw coverage.

### Outcomes

The primary outcome of this study was the amount of AOB closure measured clinically as the vertical separation between corresponding maxillary and mandibular central incisors using a digital caliper. The secondary outcomes included the amount of maxillary posterior teeth intrusion measured as the change in the distance between molar tri-furcation or premolar center (midpoint between buccal cusp tip and root apex) to the Frankfurt Horizontal plane (FH). Mandibular posterior teeth intrusion was measured as the change in the distance between molar bi-furcation or premolar center to the mandibular plane. Changes in tip and torque of intruded teeth were measured as the change in the angle between a tooth long axis and the FH and the mid-sagittal plane respectively. A three-dimensional analysis on InVivo Dental software version 5.3r (Anatomage, San Jose, CA, USA) was used to measure the rest of the secondary outcomes. Pooling of the measurements was done for all the secondary outcomes to facilitate interpretation and comparison to previous reports.

### Sample size calculation

PS calculator version 3.1.2. was used for sample size calculation. In a previous study [10], with similar methodology to the MIC group, the response was normally distributed with standard deviation of 1.18. If the true difference between the groups was estimated to be 2 mm, we needed to study 14 patients. The Type I error probability associated with this test is 0.05 and the power of the study is 80%. Oversampling was done to include 22 patients; 11 per group.

### Randomization and blinding

Simple randomization was done using Microsoft Office Excel 2019. The random sequence was generated with a 1:1 allocation ratio using (RAND) function. Numbers from 1 to 22 were written on opaque papers, folded 4 times and placed in sealed opaque envelopes. The envelopes were kept with the clinic secretary, who was not acquainted with the difference in interventions. On the day of appliance installation, each patient randomly picked an envelope. The secretary then tells the primary investigator to which group this patient should belong.

The data-analyst was blinded, as the raw data was sent designating groups 1 and 2, without explaining treatment details. The outcome assessor was blinded during taking the clinical measurements of the AOB, and was partially blinded while doing the radiographic measurements, because the radiographs showed the number of miniscrews in each patient. Blinding of the principal investigator was not possible as she was the one who did all the clinical procedures.

### Statistical methods

Statistical analysis was performed with Statistical Package for the Social Sciences (SPSS) version 20 (SPSS Inc., Chicago) for windows. *Shapiro-Wilk test of normality* was used to test the normality hypothesis for all quantitative variables. Mostly the variables were found to be normally distributed allowing the use of parametric tests. *Paired sample t test* and *independent sample t test* were applied for comparing the pre-post changes within and across the groups, respectively. Significance level is considered at *P* < 0.05. Intra- and inter-observer reliability analysis was done using concordance correlation coefficient including 95% confidence interval.

## Results

### Participant’s flow

**Fig. 2:**
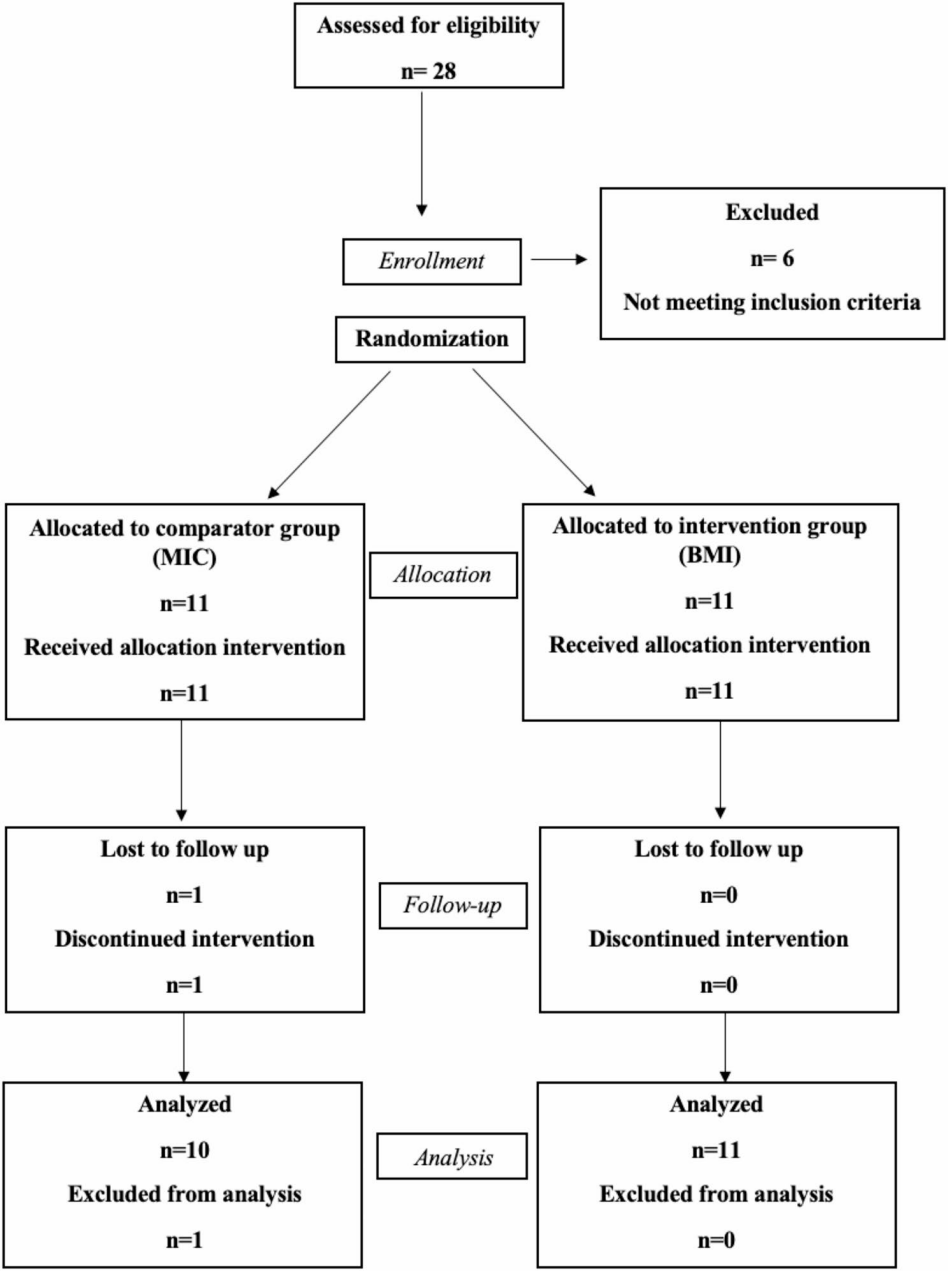
CONSORT participant flow diagram

Twenty-two patients were originally randomized into the two groups. One patient dropped out from the MIC group due to unplanned pregnancy and inability to follow-up. So, finally, eleven patients were included in the BMI group and ten patients in the MIC group. Figure 2 shows the CONSORT participant flow diagram. Recruitment started on 20th of September,2020 and ended on 15th of February,2021. Follow-up continued till 10th of January,2022. The trial was ended after 6 months of intrusion for each patient.

### Baseline characteristics

The sample included in the study had similar pre-treatment characteristics, as they all followed the aforementioned eligibility criteria. The randomized clinical trial methodology ensured that each patient had an equal chance of joining either group. Table 2 shows the demographic characteristics and comparison at baseline of the included participants in both groups, which showed baseline similarity that validates the comparison.

**Table 2:** Comparison of baseline characteristics between both groups

Variable	Group	%	Mean	SD	SEM	95% CI	
						Lower	Upper
*Demographic characteristics*							
**Gender**							
Males (%)	C	40					
	I	36.3					
Females (%)	C	60					
	I	63.7					
**Age (y)**	C		20.02	2.31	0.73	18.36	21.68
	I		19.67	2.15	0.68	18.13	21.21
*Clinical characteristics*							
**Open bite (mm)**	C		4.99	1.43	0.45	3.97	6.01
	I		6.70	1.86	0.59	5.37	8.03
**SN/MP (∘)**	C		43.21	4.98	1.58	39.64	46.77
	I		42.29	5.95	1.88	38.03	46.54

Y: years, SNMP: mandibular plane angle to SN plane,

C: comparator group, I: intervention group, SD: standard deviation,

CI: confidence interval

### Reliability analysis

The concordance correlation coefficient exceeded 0.9 for all measurements, which reflected excellent intra- and inter-observer reliability. Hence, the analysis can be considered reliable.

### Anterior open bite closure and intrusion measurements

**Fig. 3:**
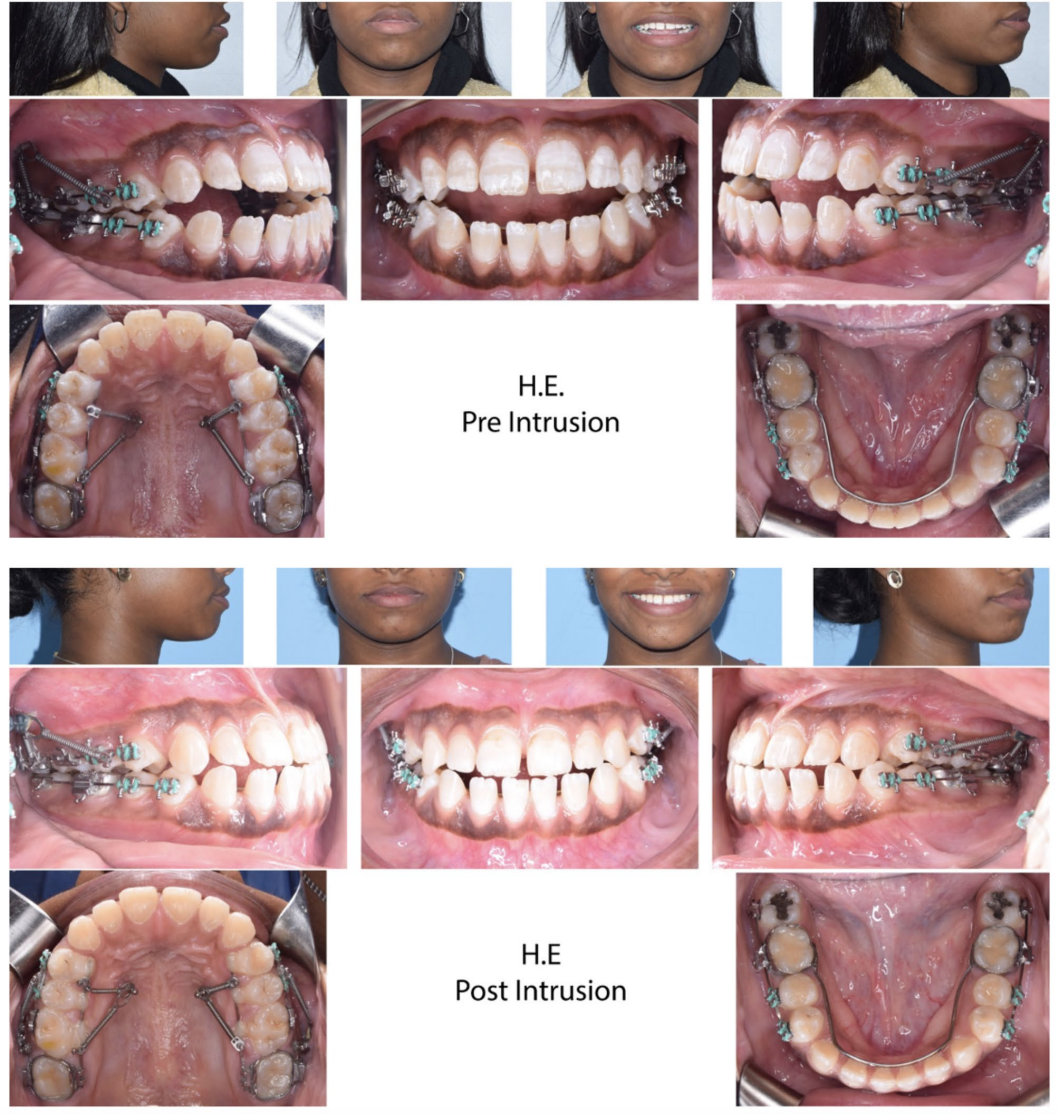
Anterior open bite closure of a patient in the MIC group

**Fig. 4:**
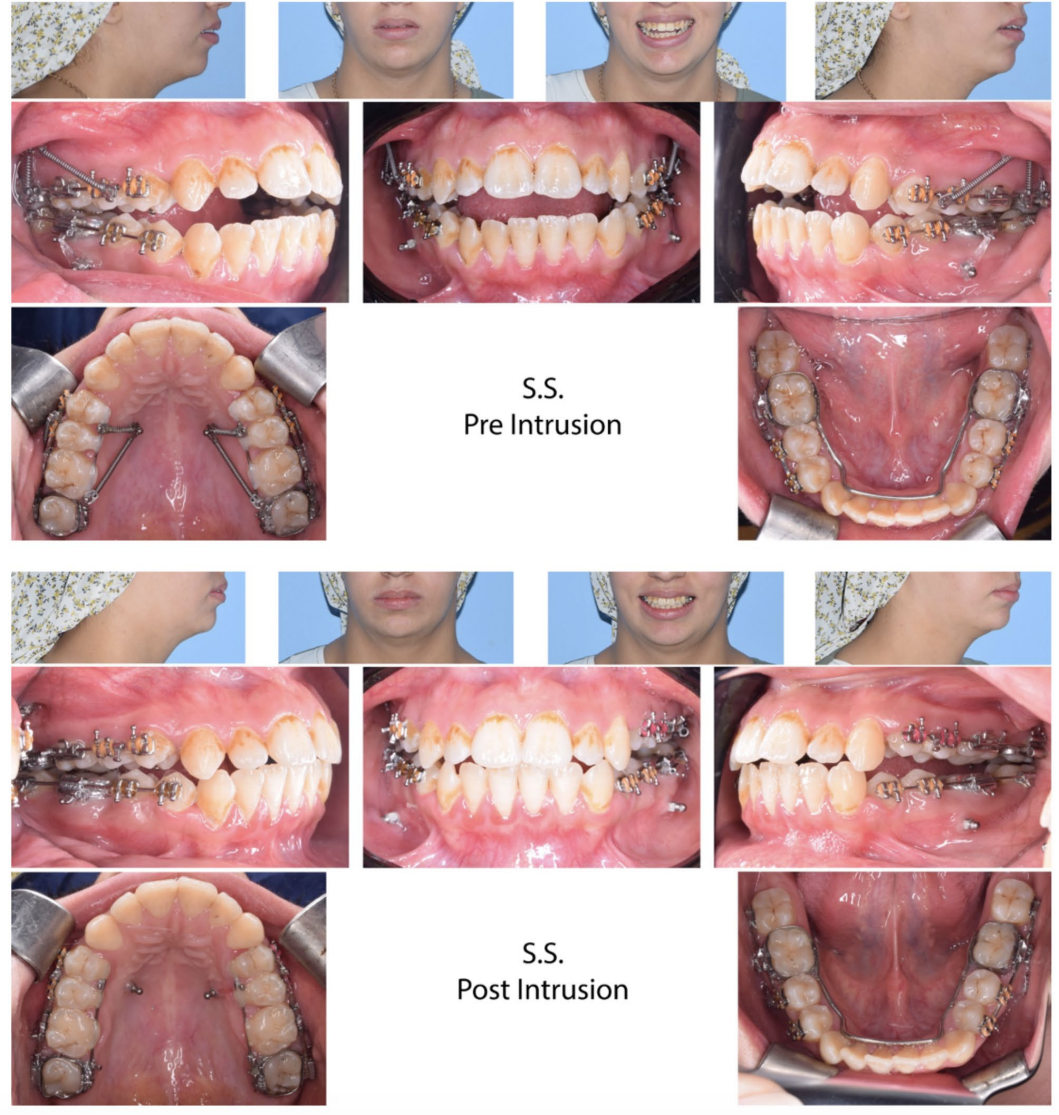
Anterior open bite closure of a patient in the BMI group

**Fig. 5:**
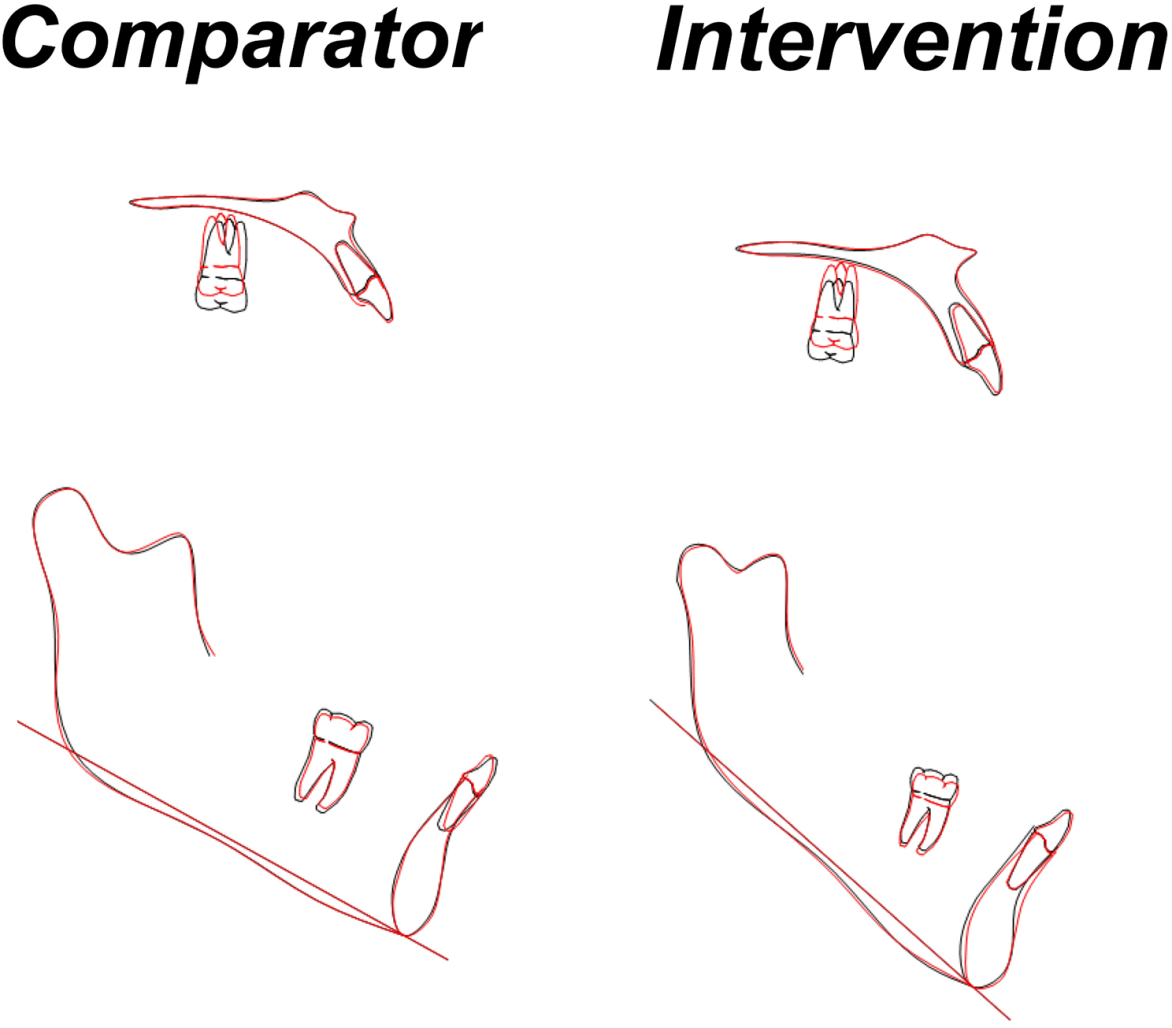
The cephalometric superimposition of pre- and post-intrusion changes of patients in both groups

There was statistically significant AOB closure in both groups, with means of 3.8 ± 0.84 mm (95% confidence interval [CI] 3.2–4.4) and 3.84 ± 1.47 mm (CI;2.8–4.9) for the MIC and BMI groups, respectively, with no significant difference between them (Table 3). Figure 3 shows the AOB closure of a patient in the MIC group, while Fig. 4 shows one in the BMI group. Maxillary posterior teeth experienced significant intrusion in both groups, with a mean of 2.89 ± 1.13 mm (CI;2.63–3.14) in the MIC group and 2.26 ± 1.62 mm (CI;1.89–2.62) in the BMI group (Table 4). The difference between the groups had a mean of 0.63 ± 0.22 mm (CI; 0.19–1.07), which showed statistical significance (Table 4). The mandibular teeth were significantly intruded as well, with a mean of 0.86 ± 0.91 mm (CI;0.65–1.06) in the BMI group and 0.33 ± 0.84 mm (CI;0.14–0.52) in the MIC group, with a statistically significant inter-group difference of 0.53 ± 0.14 mm (CI;0.25–0.8) (Table 5). There was insignificant change in tip or torque of maxillary and mandibular posterior teeth during intrusion (Tables 6 and 7). The cephalometric super-impositions of pre- and post-intrusion changes are presented in Fig. 5.

**Table 3:** Descriptive and comparative statistics of anterior open bite closure

W	MIC group							BMI group							Inter-group differences			
	Mean (mm)	SD (mm)	MD (mm)	SD (mm)	95% CI		*P*-Value	Mean (mm)	SD (mm)	MD (mm)	SD (mm)	95% CI		*P*-Value	MD (mm)	95% CI		*P*-Value
					Lower (mm)	Upper (mm)						Lower (mm)	Upper (mm)			Lower (mm)	Upper (mm)	
W2	4.361	1.27	0.329	0.29	0.120	0.538	0.0061	6.379	1.75	0.322	0.12	0.239	0.405	0.0000	-0.01	-0.22	0.20	0.9447
pre	4.690	1.43						6.701	1.86									
W4	4.113	1.29	0.577	0.32	0.351	0.803	0.0003	5.842	1.86	0.859	0.35	0.611	1.107	0.0000	0.28	-0.03	0.59	0.0738
pre	4.690	1.43						6.701	1.86									
W6	3.786	1.34	0.904	0.40	0.615	1.193	0.0001	5.544	1.78	1.157	0.37	0.896	1.418	0.0000	0.25	-0.11	0.62	0.1593
pre	4.690	1.43						6.701	1.86									
W8	3.426	1.26	1.264	0.49	0.910	1.618	0.0000	5.249	1.78	1.452	0.60	1.024	1.880	0.0000	0.19	-0.33	0.70	0.4537
pre	4.690	1.43						6.701	1.86									
W10	3.137	1.34	1.553	0.52	1.180	1.926	0.0000	4.839	1.78	1.862	0.67	1.380	2.344	0.0000	0.31	-0.26	0.88	0.2665
pre	4.690	1.43						6.701	1.86									
W12	2.827	1.32	1.863	0.64	1.403	2.323	0.0000	4.413	1.83	2.288	0.75	1.751	2.825	0.0000	0.42	-0.23	1.08	0.1907
pre	4.690	1.43						6.701	1.86									
W14	2.461	1.45	2.229	0.82	1.646	2.812	0.0000	4.067	1.76	2.634	0.75	2.100	3.168	0.0000	0.41	-0.33	1.14	0.2616
pre	4.690	1.43						6.701	1.86									
W16	2.167	1.44	2.523	0.86	1.911	3.135	0.0000	3.730	1.76	2.971	0.79	2.404	3.538	0.0000	0.45	-0.33	1.22	0.2402
pre	4.690	1.43						6.701	1.86									
W18	1.759	1.36	2.931	0.82	2.347	3.515	0.0000	3.551	1.86	3.150	0.95	2.472	3.828	0.0000	0.22	-0.61	1.05	0.5866
pre	4.690	1.43						6.701	1.86									
W20	1.428	1.43	3.262	0.75	2.725	3.799	0.0000	3.352	1.90	3.349	1.03	2.615	4.083	0.0000	0.09	-0.76	0.93	0.8312
pre	4.690	1.43						6.701	1.86									
W22	1.085	1.38	3.605	0.79	3.040	4.170	0.0000	3.044	2.05	3.657	1.26	2.758	4.556	0.0000	0.05	-0.93	1.04	0.9130
pre	4.690	1.43						6.701	1.86									
W24	0.886	1.39	3.804	0.84	3.205	4.403	0.0000	2.858	2.23	3.843	1.47	2.791	4.895	0.0000	0.04	-1.09	1.16	0.9427
pre	4.690	1.43						6.701	1.86									

W: week, SD: standard deviation, MD: mean difference, CI: confidence interval, *P*≥ 0.05: non-significant, *P*≤ 0.05: significant

MIC group: Maxillary Intrusion with Consolidation of mandibular posterior segments

BMI group: Bi-Maxillary Intrusion group (intrusion of maxillary and mandibular posterior segments)

**Table 4:** Descriptive and comparative statistics of maxillary posterior teeth intrusion measurements

Variable	MIC group						BMI group						Inter-group differences				
	Mean (mm)	SD (mm)	SEM (mm)	95% CI		*P* value	Mean (mm)	SD (mm)	SEM (mm)	95% CI		*P* value	MD (mm)	SD (mm)	95% CI		*P* Value
				Lower (mm)	Upper (mm)					Lower (mm)	Upper (mm)				Lower (mm)	Upper (mm)	
UR4-FH	3.10	1.56	0.49	1.98	4.22	0.00014	2.52	1.77	0.56	1.25	3.78	0.00147	-0.59	0.75	-2.15	0.98	0.44265
UR5-FH	3.54	1.25	0.40	2.64	4.43	0.00001	2.27	2.13	0.67	0.74	3.80	0.00831	-1.27	0.78	-2.91	0.38	0.12307
UR6-FH	3.16	0.85	0.27	2.55	3.76	0.00000	2.09	1.82	0.58	0.78	3.39	0.00554	-1.07	0.64	-2.40	0.27	0.10974
UR7-FH	2.48	0.89	0.28	1.85	3.12	0.00001	2.11	1.20	0.38	1.26	2.97	0.00034	-0.37	0.47	-1.36	0.62	0.44362
UL4-FH	2.61	1.47	0.47	1.56	3.67	0.00033	2.46	2.07	0.65	0.98	3.94	0.00447	-0.15	0.80	-1.84	1.53	0.85000
UL5-FH	2.83	1.11	0.35	2.04	3.63	0.00002	2.50	1.56	0.49	1.38	3.61	0.00068	-0.34	0.61	-1.61	0.93	0.58463
UL6-FH	2.77	0.75	0.24	2.24	3.31	0.00000	2.08	1.50	0.47	1.01	3.16	0.00176	-0.69	0.53	-1.81	0.42	0.20858
UL7-FH	2.60	0.92	0.29	1.93	3.26	0.00001	2.02	1.14	0.36	1.20	2.83	0.00035	-0.58	0.46	-1.56	0.40	0.22810
Pooled Mx. intrusion	2.89	1.13	0.13	2.63	3.14	0.00000	2.26	1.62	0.18	1.89	2.62	0.00000	-0.63	0.22	-1.07	-0.19	0.00487

UR: upper right, UL: upper left, FH: Frankfurt Horizontal plane, SD: standard deviation, SEM: standard error mean,

MD: mean difference, CI: confidence interval, *P*≥ 0.05: non-significant, *P*≤ 0.05: significant

MIC group: Maxillary Intrusion with Consolidation of mandibular posterior segments

BMI group: Bi-Maxillary Intrusion group (intrusion of maxillary and mandibular posterior segments)

**Table 5:** Descriptive and comparative statistics of mandibular posterior teeth intrusion measurements

Variable	MIC group						BMI group						Inter-group differences				
	Mean (mm)	SD (mm)	SEM (mm)	95% CI		*P* value	Mean (mm)	SD (mm)	SEM (mm)	95% CI		*P* value	MD (mm)	SD (mm)	95% CI		*P* Value
				Lower (mm)	Upper (mm)					Lower (mm)	Upper (mm)				Lower (mm)	Upper (mm)	
LR4-MP	0.08	0.82	0.26	-0.50	0.67	0.75056	0.66	0.55	0.18	0.26	1.06	0.00442	0.58	0.31	-0.08	1.23	0.08232
LR5-MP	0.41	0.84	0.26	-0.19	1.00	0.15789	0.83	0.74	0.23	0.30	1.36	0.00616	0.42	0.35	-0.32	1.16	0.24580
LR6-MP	0.50	0.70	0.22	0.00	1.00	0.05121	1.38	1.15	0.36	0.56	2.20	0.00418	0.88	0.42	-0.01	1.78	0.05216
LR7-MP	0.91	0.97	0.31	0.22	1.61	0.01530	0.79	0.96	0.31	0.10	1.48	0.02972	-0.13	0.43	-1.03	0.78	0.77222
LL4-MP	0.16	0.81	0.26	-0.42	0.75	0.53930	0.74	0.65	0.20	0.27	1.20	0.00570	0.57	0.33	-0.12	1.26	0.09844
LL5-MP	-0.05	0.63	0.20	-0.50	0.40	0.81451	0.58	0.54	0.17	0.19	0.96	0.00829	0.62	0.26	0.07	1.17	0.02857
LL6-MP	0.38	0.91	0.29	-0.27	1.03	0.22362	1.09	1.27	0.40	0.18	2.00	0.02373	0.72	0.49	-0.32	1.76	0.16401
LL7-MP	0.24	0.93	0.29	-0.42	0.91	0.42754	0.78	1.14	0.36	0.03	1.60	0.05839	0.54	0.47	-0.44	1.52	0.26214
Pooled Md. intrusion	0.33	0.84	0.09	0.14	0.52	0.00076	0.86	0.91	0.10	0.65	1.06	0.00000	0.53	0.14	0.25	0.80	0.00000

LR: lower right, LL: lower left, MP: mandibular plane, SD: standard deviation, SEM: standard error mean, CI: confidence interval

*P*≥ 0.05: non-significant, *P*≤ 0.05: significant

MIC group: Maxillary Intrusion with Consolidation of mandibular posterior segments

BMI group: Bi-Maxillary Intrusion group (intrusion of maxillary and mandibular posterior segments)

**Table 6:** Descriptive and comparative statistics of torque and tip change of maxillary posterior teeth during intrusion

Variable	MIC group						BMI group						Inter-group differences				
	Mean (∘)	SD(∘)	SEM (∘)	95% CI		*P* value	Mean (∘)	SD(∘)	SEM (∘)	95% CI		*P* value	MD (∘)	SD (∘)	95% CI		*P* Value
				Lower (∘)	Upper (∘)					Lower (∘)	Upper (∘)				Lower (∘)	Upper (∘)	
UR4/MSP	0.36	1.81	0.57	-0.93	1.65	0.54457	0.97	2.75	0.87	-0.99	2.94	0.29208	-0.61	1.04	-2.80	1.57	0.56313
UR5/MSP	-0.02	3.71	1.17	-2.68	2.64	0.98811	0.82	3.53	1.12	-1.70	3.35	0.47950	-0.84	1.62	-4.24	2.56	0.60999
UR6/MSP	-1.85	2.62	0.83	-3.73	0.03	0.05278	1.43	5.65	1.79	-2.61	5.47	0.44410	-3.28	1.97	-7.41	0.86	0.11320
UR7/MSP	2.26	2.12	0.67	0.74	3.77	0.00832	0.09	5.58	1.77	-3.90	4.09	0.95826	2.16	1.89	-1.81	6.13	0.26761
UL4/MSP	0.67	2.59	0.82	-1.19	2.53	0.43461	0.74	4.95	1.57	-2.80	4.28	0.64762	-0.07	1.77	-3.78	3.64	0.96929
UL5/MSP	0.62	2.80	0.89	-1.38	2.63	0.49913	0.65	4.36	1.38	-2.46	3.77	0.64594	-0.03	1.64	-3.47	3.41	0.98511
UL6/MSP	-0.44	2.76	0.87	-2.41	1.54	0.62722	2.47	5.74	1.82	-1.64	6.57	0.20730	-2.91	2.01	-7.14	1.33	0.16637
UL7/MSP	-0.23	3.02	0.96	-2.39	1.93	0.81824	-1.46	5.58	1.77	-5.45	2.54	0.43099	1.23	2.01	-2.99	5.45	0.54779
**Pooled Mx torque change**	0.17	2.83	0.32	-0.46	0.80	0.58770	0.72	4.78	0.53	-0.35	1.78	0.18417	-0.54	0.62	-1.77	0.68	0.38272
UR4/FH	-2.46	6.37	2.01	-7.01	2.10	0.25387	-1.27	6.29	1.99	-5.76	3.23	0.53938	-1.19	2.83	-7.13	4.76	0.67983
UR5/FH	-0.31	4.65	1.47	-3.64	3.02	0.83823	0.70	7.32	2.32	-4.54	5.94	0.76869	-1.01	2.74	-6.77	4.75	0.71678
UR6/FH	0.20	5.37	1.70	-3.64	4.05	0.90752	0.65	8.45	2.67	-5.39	6.69	0.81264	-0.45	3.17	-7.10	6.20	0.88879
UR7/FH	-0.34	4.74	1.50	-3.73	3.05	0.82616	-0.31	10.62	3.36	-7.90	7.29	0.92890	-0.03	3.68	-7.75	7.69	0.99336
UL4/FH	2.71	8.07	2.55	-3.06	8.47	0.31651	1.00	6.39	2.02	-3.57	5.57	0.63134	1.70	3.25	-5.13	8.54	0.60724
UL5/FH	-0.33	5.42	1.71	-4.21	3.54	0.85028	2.64	9.00	2.85	-3.81	9.08	0.37874	-2.97	3.32	-9.95	4.01	0.38348
UL6/FH	-0.38	6.93	2.19	-5.33	4.58	0.86722	0.78	4.94	1.56	-2.76	4.31	0.63069	-1.16	2.69	-6.81	4.50	0.67299
UL7/FH	-2.63	6.85	2.17	-7.53	2.27	0.25530	3.38	5.31	1.68	-0.42	7.18	0.07507	-6.01	2.74	-11.77	-0.25	0.04169
**FH/UR post. segment line**	-0.56	2.44	0.77	-2.30	1.19	0.48873	-0.02	1.27	0.40	-0.93	0.89	0.96720	-0.54	0.87	-2.37	1.29	0.54265
**FH/UL post. segment line**	-0.14	2.90	0.92	-2.22	1.93	0.87945	-1.19	3.01	0.95	-3.34	0.96	0.24226	1.05	1.32	-1.73	3.82	0.43797

UR: upper right, UL: upper left, MSP: mid-sagittal plane, FH: Frankfurt Horizontal plane, SD: standard deviation,

SEM: standard error mean, CI: confidence interval, *P*≥ 0.05: non-significant, *P*≤ 0.05: significant

MIC group: Maxillary Intrusion with Consolidation of mandibular posterior segments

BMI group: Bi-Maxillary Intrusion group (intrusion of maxillary and mandibular posterior segments)

**Table 7:** Descriptive and comparative statistics of mandibular posterior teeth torque change during consolidation/intrusion

Variable	MIC group						BMI group						Inter-group differences				
	Mean (∘)	SD(∘)	SEM (∘)	95% CI		*P* value	Mean (∘)	SD(∘)	SEM (∘)	95% CI		*P* value	MD (∘)	SD (∘)	95% CI		*P* Value
				Lower (∘)	Upper (∘)					Lower (∘)	Upper (∘)				Lower (∘)	Upper (∘)	
LR4/MSP	-0.45	1.91	0.60	-1.81	0.92	0.47704	1.83	4.58	1.45	-1.45	5.11	0.23774	-2.28	1.57	-5.58	1.02	0.16350
LR5/MSP	-0.55	0.97	0.31	-1.24	0.15	0.10812	1.41	2.48	0.79	-0.37	3.19	0.10646	-1.96	0.84	-3.73	-0.18	0.03229
LR6/MSP	-0.09	2.48	0.78	-1.86	1.68	0.91008	-1.13	2.83	0.90	-3.16	0.90	0.23886	1.04	1.19	-1.46	3.54	0.39411
LR7/MSP	-0.14	2.39	0.76	-1.85	1.57	0.86023	0.35	4.33	1.37	-2.75	3.45	0.80400	-0.49	1.56	-3.77	2.80	0.75911
LL4/MSP	-0.62	2.10	0.66	-2.13	0.88	0.37314	2.08	3.44	1.09	-0.38	4.54	0.08846	-2.70	1.27	-5.38	-0.02	0.04831
LL5/MSP	1.84	1.63	0.52	0.67	3.01	0.00608	1.89	3.43	1.08	-0.56	4.34	0.11479	-0.05	1.20	-2.57	2.47	0.96526
LL6/MSP	1.86	2.88	0.91	-0.20	3.92	0.07152	-1.16	2.31	0.73	-2.82	0.49	0.14577	3.03	1.17	0.57	5.48	0.01851
LL7MSP	-1.23	2.77	0.88	-3.21	0.75	0.19357	0.59	4.35	1.38	-2.52	3.71	0.67680	-1.82	1.63	-5.25	1.60	0.27853
Pooled Md. Torque change	0.08	2.38	0.27	-0.45	0.61	0.77060	0.73	3.62	0.41	-0.07	1.54	0.07432	-0.65	0.48	-1.61	0.30	0.17867

LR: lower right, LL: lower left, MP: mandibular plane, SD: standard deviation, SEM: standard error mean, CI: confidence interval

MD: mean difference,*P*≥ 0.05: non-significant, *P*≤ 0.05: significant

MIC group: Maxillary Intrusion with Consolidation of mandibular posterior segments

BMI group: Bi-Maxillary Intrusion group (intrusion of maxillary and mandibular posterior segments)

### Miniscrews’ failure rate

The failure rate of miniscrews was 1 out of 42 for the infra-zygomatic region (2.4%), zero for the palatal miniscrews (0%) and 4 out of 22 for the mandibular buccal screws (18%). The overall percentage of failure was 4.7% (5 out of 106 miniscrews).

### Harms

Some patients experienced discomfort after placement of the miniscrews. They were given strict oral hygiene measures to minimize inflammation around the screws and promote their stability. Patients were also instructed to do blowing exercises to avoid soft tissue coverage around the infra-zygomatic screws.

## Discussion

The SOB represents a complex orthodontic problem [1–3]. The increased use of skeletal anchorage has widened the range of conservative treatment options for such malocclusion, enabling AOB closure by posterior teeth intrusion [10, 15, 19]. However, the available literature reports voids in the evidence and lack of properly designed studies investigating the maxillary and/or mandibular posterior segments’ intrusion [8, 9, 12, 14]. The diagnostic shortcomings of AOB malocclusion might as well contribute to the distortion of evidence due to lack of understanding of the contributing factors of AOB and their phenotypic distinction [2, 20]. Previous studies have validated the skeletal-anchorage-supported maxillary posterior segments’ intrusion in the treatment of SOB [10, 15, 21]. Nevertheless, compensatory extrusion of the mandibular posterior segments took place simultaneous to this, resulting in unsatisfactory results of AOB closure [10, 15]. Therefore, the methodology of this study was designed to compare maxillary posterior segments’ intrusion with consolidation versus active intrusion of the mandibular posterior segments in treatment of SOB in adult patients.

The consolidation of the mandibular posterior segments by means of a lingual arch and a rigid stainless steel archwire was done in both groups. Hence the passive consolidation in the MIC group was compared to the active intrusion by miniscrews in the BMI group. The lingual arch offered an extra technical advantage in the BMI group, which was the ability to add lingual crown/buccal root torque before its cementation to overcome the buccal rolling due to the buccally applied intrusive force.

The mandibular posterior segments experienced statistically significant intrusion in the BMI group with a mean of 0.86 mm, which indicates the success of miniscrews to actively intrude those segments. On the other hand, the MIC group showed minimal intrusion with a mean of 0.33 mm, which might be due to the tongue pressure on the relieved lingual arch. This proves that the combination of the lingual arch and the rigid archwire effectively prevented the extrusion of the mandibular posterior segments during maxillary posterior segments’ intrusion. The difference between the groups had a mean value of 0.53 mm, which despite showing statistical significance, was not considered clinically significant. This judgment was based on the fact that the difference did not affect the AOB closure, and it only occurred in the pooled result due to the increased sample size, which decreases the p-value even with a numerically small mean difference. *Xun et al.* [16] reported similar findings of lower molars’ intrusion with a mean of 1.2 mm resulting in 4.2 mm of AOB closure.

The maxillary posterior segments were significantly intruded in both groups with means of 2.89 and 2.26 mm in the MIC and BMI groups, respectively. These results are comparable to most of the previous studies [10, 15, 19, 22]. That is to say that the mean amount of maxillary posterior teeth intrusion falls in the range from 2 to 3 mm [21, 23, 24]. *Akan et al.* [11] and *Akbaydogan and Akin* [25] reported greater means for maxillary posterior teeth intrusion, of 3.37 and 4 mm, respectively and consequently greater amounts of AOB closure of 4.79 mm for the former and 5.81 mm for the latter. These improved outcomes could be attributed to the longer study duration, as well as their use of maxillary occlusal splints, adding more intrusive force to the molars “bite block effect” [26]. The use of occlusal splints was avoided in the current study as it was considered a confounder during AOB closure. The difference between the MIC and BMI groups in this study was 0.63 mm, which showed statistical significance of the pooled result, but not in individual teeth measurements, and did not affect the AOB closure. Therefore, it was considered clinically insignificant.

During AOB closure, two patients had interferences at the canine teeth. The interferences were located by an articulating paper and selective grinding was done in order to allow the true amount of AOB closure to be expressed. A similar finding was previously reported [10, 15] and was accused of hindering the effect of posterior teeth intrusion on AOB closure.

The use of CBCT in the current study extended the capacity to analyze the treatment effects more meticulously. The choice of valid and reliable landmarks for intrusion measurements relying on molar bi/trifurcation or premolar center to the Frankfurt Horizontal plane was intended to offer substantial accuracy as compared to the 2-dimensional measurements reported previously. The ability to customize each landmark identification on the best view, and then refining it on the other views was exploited for greater efficiency. Other studies used lateral cephalometric radiographs [11, 16, 22, 24] and measured intrusion from the mesio-buccal cusp or cervical margin of first molars to the palatal plane [15, 16, 19]. Such practice would presumably suffer some inaccuracies due to the inherent difficulty in placing these landmarks on 2-dimensional radiographs [27] and the possible clockwise rotation of the palatal plane consequent to posterior intrusion [16, 23, 24], which renders it a weak reference plane in these cases. The advancements in technology allowed better information to be derived from 3-dimensional analysis with reduced exposure parameters to the patients [28, 29]. Furthermore, the great intra- and inter-observer reliability of the customized analysis used in this study corroborated the immense benefit of utilizing CBCT. The decision to take CBCT was based on a balance of clinical necessity and radiation safety, which is essentially important in cases with complex malocclusions like the skeletal open bite.

The findings of this study highlight the value of methodologically sound research and its ability to prove or refute assumptions that could have been made based on the results of different or low-quality articles. The findings of *Xun et al.* [16] suggest that mandibular posterior segments’ intrusion would result in greater amount of AOB closure when compared to those of *Akl et al.* [10] However, upon applying randomization, allocation concealment and blinding when possible, this assumption was disputed and the true difference between the groups was proven to be non-existent.

The overall failure rate of miniscrews used in the current study is considered low, with only 4.7% as compared to the average of 13.5% reported in a recent systematic review [30]. This can be attributed to the homogenous sample used, the choice of sites with good stability (infra-zygomatic and palatal sites) [31] and the strict disinfection measures followed during miniscrew placement. Additionally, the application of low continuous forces may have contributed to the low failure rate. *Scheffler et al.* [15] was the only study to report miniscrew failure rate, where one infra-zygomatic miniscrew and another palatal one failed. However, this study had a retrospective design, included patients who received miniplates and miniscrews, as well as growing and non-growing subjects. Such heterogeneity kind of invalidates the comparison to the results of the current study.

Based on the aforementioned results and their interpretation, it can be stated that maxillary posterior segments’ intrusion can be considered a valid conservative treatment modality for moderate skeletal open bite in adult patients. Careful planning of the miniscrews’ position and elimination of interferences is essential for successful AOB closure. It can as well be elucidated that there might not be a strict indication for mandibular posterior segments’ intrusion as long as they are effectively consolidated.

### Limitations and generalizability

The 6-months duration of intrusion; although similar to many previous reports, could be considered a limitation in the current study, as other studies with longer observation periods of 8–9 months reported greater amounts of intrusion and anterior open bite closure.

Despite having been conducted in a single center and by a single primary investigator, all attempts to reduce bias were considered in the current study, including randomization, allocation concealment, and blinding whenever possible. The similarity of these results to previous reports might indicate their possible generalization.

## Conclusions

Within the limitations of the current study, the following can be concluded:

1. Skeletal-anchorage supported intrusion of maxillary posterior segments with consolidation or active intrusion of the mandibular posterior segments was a successful conservative treatment modality for skeletal open bite in adult patients, yielding comparable amounts of anterior open bite closure.
2. Active intrusion of the mandibular posterior segments might not be needed for anterior open bite closure, as long as they are efficiently consolidated via a lingual arch and a rigid segmented archwire during maxillary posterior segments’ intrusion.

## Data Availability

No datasets were generated or analysed during the current study.

## Abbreviations

AOB: Anterior open bite
SOB: Skeletal open bite
MIC: Maxillary Intrusion with Consolidation of mandibular buccal segments
BMI: Bi-Maxillary Intrusion
